# The Shoulder Function Index (SFInX): a clinician-observed outcome measure for people with a proximal humeral fracture

**DOI:** 10.1186/s12891-015-0481-x

**Published:** 2015-02-18

**Authors:** Alexander TM van de Water, Megan Davidson, Nora Shields, Matthew C Evans, Nicholas F Taylor

**Affiliations:** Department of Physiotherapy, School of Allied Health and LASER (La Trobe Sports, Exercise and Rehabilitation), La Trobe University, Bundoora, Victoria 3086 Australia; Department of Allied Health, Northern Health, Bundoora, Victoria 3083 Australia; Melbourne Orthopeadic Group, Windsor, Victoria 3181 Australia; Allied Health Clinical Research Office, Eastern Health, Box Hill, Victoria 3128 Australia

**Keywords:** Shoulder fractures, Rehabilitation, Outcome assessment (Health Care), Shoulder function index, Rasch analysis, Content and structural validity

## Abstract

**Background:**

Proximal humeral fractures are amongst the most common fractures. Functional recovery is often slow and many people have ongoing disability during activities of daily life. Unidimensional measurement of activity limitations is required to monitor functional progress during rehabilitation. However, currentshoulder measures are multidimensional incorporating constructs such as activities, range of motion and pain into a single scale. Psychometric information of these measures is scarce in this population, and indicate measurement issues with reliability. Therefore, the aim was to develop the clinician-observed Shoulder Function Index (SFInX), a unidimensional, interval-level measure of ‘shoulder function’ based on actual performance of activities, reflecting activity limitations following a proximal humeral fracture.

**Methods:**

An outcome measure development study was performed including item generation (existing shoulder measures, focus groups) and item selection (selection criteria, importance and feasibility ratings, pilot testing, Rasch analysis). Clinicians (n=15) and people with a proximal humeral fracture (n=13) participated in focus groups. Items were pilot tested (n=12 patients) and validated in a Rasch study. The validation study sample (n=92, 86% female) were recruited between 5 and 52 weeks post-fracture and had a mean age of 63.5 years (SD13.9). Measurements at recruitment and 6 and 7 weeks later were taken in three public metropolitan hospitals or during home visits. Raw SFInX data were analysed with WINSTEPS v3.74 using polytomous Rasch models.

**Results:**

From 282 generated items, 42 items were selected to be rated by clinicians and patients; 34 items were pilot tested and 16 items were included for Rasch analysis. The final SFInX, developed with the Partial Credit Model, contains 13 items and has the response categories: ‘unable’, ‘partially able’ and ‘able’. It is unidimensional measuring ‘shoulder function’, and can measure from early functional use (drinking from a cup) to independence around the house (lifting items above head, carrying heavy items).

**Conclusions:**

The SFInX is a promising outcome measure of shoulder function for people with a proximal humeral fracture. It has content relevant to patients and clinicians, is unidimensional and feasible for use in clinical and home settings. In its current form, the SFInX is ready for further psychometric evaluation, and for subsequent use in clinical settings and research.

**Electronic supplementary material:**

The online version of this article (doi:10.1186/s12891-015-0481-x) contains supplementary material, which is available to authorized users.

## Background

Fractures of the proximal humerus are one of the most common limb fractures [1,2] that mainly occur in people over the age of 50 years [3-5]. Because of the aging population, the incidence of the proximal humeral fractures is expected to increase over the next decades [6].

Functional recovery after a proximal humeral fracture is often slow and many patients experience ongoing disability during activities of daily life [7-9]. The first weeks after injury are characterised by reduced arm function and severe pain. Typically, the active phase of rehabilitation commences between 2 and 6 weeks post-fracture [7,10,11]. Functional improvements are expected in the following months with gradual return to self-care and daily activities. Between 6 to 12 months, most people can perform their activities for daily living, but often with a certain degree of difficulty. The ongoing disability after a proximal humeral fracture is often experienced as limitations in performing activities [7-9]. According to the *International Classification of Functioning, Disability and Health* (ICF) framework, activity limitations are difficulties an individual may have in executing activities or tasks [12]. Patients may be limited in activities such as placing objects into high cupboards, washing their lower back, or carrying items. Such limitations in activities might reduce independence and potentially influence level of participation in normal societal roles.

If activity limitations are important to people with a proximal humeral fracture, it is important to be able to measure this construct, so that appropriate interventions can be chosen for these patients and functional progress can be monitored [13]. This requires a functional outcome measure that is unidimensional (measures the single construct of activity limitations), psychometrically sound, relevant to the patient and clinically feasible.

None of the currently used outcome measures in people with a proximal humeral fracture [14] measure the single construct of activity limitations. For example, the clinician-administered Constant Score and American Shoulder Elbow Surgeons (ASES) Shoulder Score [15] assess components of pain, ‘function’ or activities, range of motion and strength and combine these in a single score. The Disabilities of the Arm, Shoulder and Hand (DASH) [16] and Oxford Shoulder Score [17] questionnaires partly assess activity limitations, but also include items related to pain/sensation and psychological factors. Incorporating multiple constructs in one outcome measure and summing their subscores into one total score may obscure outcomes in the different domains. Although clinicians may look at the individual items to determine this for individual patients, this reduces the utility of the instrument for clinical and research purposes. To measure activity limitations in people recovering from a proximal humeral fracture, a unidimensional outcome measure is required.

In addition to current shoulder outcome measures having a multidimensional structure, there is little and limited psychometric information for these measures in people with a proximal humeral fracture, particularly during the active phase of rehabilitation [14,18-21]. Also, the information that is available suggests that existing scales may have problems with relatively wide limits of agreement (for example, ±15% of total scores for the DASH) and structural validity (for example, inclusion of multiple constructs and redundant items) [11,15-18].

Therefore, there is a need to develop a unidimensional outcome measure with sound psychometric properties that can evaluate activity limitations in people with a proximal humeral fracture. The main aim of this study was to develop the Shoulder Function Index (SFInX). During its development, it underwent Rasch analysis, ensuring it is unidimensional, measuring the construct of ‘shoulder function’ which is scored on a linear, interval-level scale.

## Methods

The construct of the new outcome measure was broadly defined as ‘shoulder function’, the ability to perform activities in which the shoulder is involved. It was developed to reflect the ‘*Activities*’ component of the *International Classification of Functioning, Disability and Health* framework [12]. The SFInX aims to reflect the abilities or limitations in activities of people recovering from a proximal humeral fracture.

Ethics approval was obtained from the relevant hospital (Eastern Health) and university (La Trobe University) human ethics committees. All participants provided written informed consent. We also obtained written informed consent from the individual whose photographic images are present in the SFInX manual (Additional file 1) to use and publish these images.

**Figure 1:**
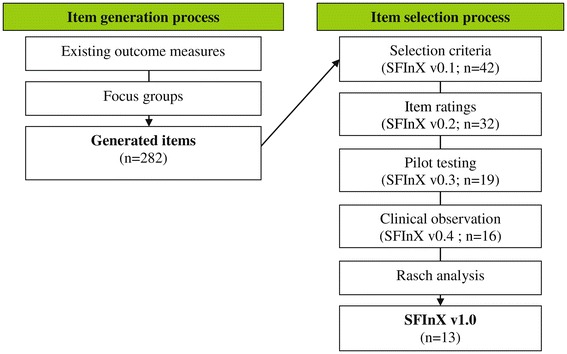
**Development process of the SFInX: stages of generation and selection of items, including the SFInX versions and number of items retained at each stage.**

Development of the SFInX was comprised of two phases: item generation and item selection. An item pool was generated from existing shoulder outcome measures and from focus groups with patients and clinicians. Then, items were selected using selection criteria, item ratings, pilot testing, clinical observation and Rasch analysis (Figure 1).

### Item generation

Items from 17 shoulder outcome measures, identified in a recent review [14], that met selection criteria (see ‘Item selection’) were placed into the item pool. Additional items were generated from four focus groups, conducted by an experienced moderator and assistant, with up to eight participants in each group. Two focus groups included people who had sustained a proximal humeral fracture in the previous year, and two focus groups included clinicians: orthopedic surgeons, physical therapists or occupational therapists. Purposive sampling [22] based on participant characteristics ensured diversity and improved discussion among the participants.

### Item selection

Item selection was comprised of four methods (Figure 1): selection based on pre-determined criteria, item ratings by focus group participants, clinical testing and Rasch analysis.

Items from the generated list were selected independently by two researchers using the pre-determined selection criteria below, and consensus was reached by discussion within the research team:

- ▪ Able to be categorised in the ICF categories of the ‘Activities’ component (for example, d4 Mobility, d5 Self-care or d6 Domestic life)
- ▪ Describe a single task/activity
- ▪ Require no or minimal equipment
- ▪ Be easy and quick to perform
- ▪ Able to be administered by health professionals from a range of disciplines

To enhance face validity further selection of these items was based on feedback from focus group participants. They were sent an item rating questionnaire to rate the importance and clinical testing feasibility (clinicians only) of the items on an 11-point Numeric Rating Scale, ranging from 0 (not at all important, or feasible) to 10 (very important, or feasible). A pre-determined arbitrary threshold of 7/10 (on average) per item was set as a guideline for further item selection.

Included items were named and defined for pilot testing. This included the descriptions of positions, instructions and scoring categories. Scoring of a person’s ability to perform an activity was defined as ‘unable’, ‘partially able’ and ‘able’.

Twelve people recovering from a proximal humeral fracture were recruited for pilot-testing of the items. This phase aimed to identify and evaluate the adequacy of the test protocol descriptions, feasibility issues, discriminatory characteristics of items, adequate reflection of ‘shoulder function’ by the items, and the perceptions of participants and researchers.

If an item was not practical, could not discriminate among people (for example, all people could perform the activity) or did not capture the construct of ‘shoulder function’, the item was altered or removed. If participants or the researchers identified that easy or difficult activities were missing, a new item was included in the pilot test list.

### Participants

For all phases of the study, people with a proximal humeral fracture were identified from a health service database and through the Emergency and Physiotherapy Departments of three metropolitan hospitals in Melbourne (Victoria, Australia). Potentially eligible participants were mailed an invitation to take part in the study and non-responders were followed up by telephone. Interested individuals were screened against eligibility criteria (Table 1). Eligible individuals were invited to attend one of the hospitals or were offered a home visit.

**Table 1:** **Selection criteria of participants**

*Inclusion criteria*	• 18 years or older
	• Isolated proximal humeral fracture, or proximal humeral fracture-dislocation with similar clinical presentation after reduction
	• Available for recruitment within one year (365 days) post-fracture
	• Any treatment received for proximal humeral fracture
	• Ability to complete English-language questionnaires and to follow simple instructions in English
	• Short Portable Mental Status Questionnaire score 6–10 (indicates intact or mildly impaired cognitive functioning)
*Exclusion criteria*	• Other serious medical issues from the trauma (e.g. hip fracture, wrist fracture, nerve lesion, traumatic brain injury, muscle ruptures)
	• Potentially confounding medical conditions (e.g. hemiplegic arm, previous shoulder surgery, re-fracture, severe rheumatoid arthritis)

Demographics and characteristics of participants of the validation study were gathered through interview and completion of short questionnaires regarding the cause of fracture, preferred/dominant side, living status [20], the Self-Administered Co-morbidity Questionnaire [23], and pre-fracture activity level (using 15 items specific to the upper extremity from the Human Activities Profile [24] with a total possible score 0 to 15). Based on X-rays, fractures were classified by an orthopaedic consultant according to three classifications systems for comprehensive description of fracture types: the Neer [25], AO [26] and Codman-Hertel [27] classifications.

### Sample size of the validation study

Guidelines set out by Linacre [28] proposed a sample size of 100 for stable estimations of item calibrations within 0.5 logits (logarithmic odds units) with 95% confidence. If a test is well targeted, that is, person abilities have a range similar to the range of difficulty of items, fewer observations are needed (*n* = 64). If a test is less adequately targeted, a larger sample is required (*n* = 144). Therefore, we hypothesised that a minimum target sample size of the validation study of *n* = 75 participants would be sufficient.

### Data analysis

#### Statistical software

For Rasch analyses WINSTEPS version 3.74 was used. WINSTEPS uses three mathematical methods to estimate the Rasch parameters. First, the unanchored person and item estimations are set to zero. Then, the Normal Approximation Algorithm method [29,30] is employed, followed by Joint Maximum Likelihood Estimation [31]. For descriptive and comparative analyses (*t*-test, *p* < 0.05) IBM Statistical Package for Social Sciences 19.0 was used.

#### Rasch analysis

We employed a comprehensive and pragmatic analysis using two different Rasch models simultaneously for comparison [32,33], the Andrich-Rasch Grouped-Rating Scale Model (GRSM) [34] and the Rasch-Masters Partial Credit Model (PCM) [35].

Rasch analysis is an iterative process, continuously cross-checking statistics, especially when items or unexpected responses have been removed. Overall test functioning and specific item and person fit to the models were checked. Guidelines by Wright and Linacre [36] were used to evaluate item and person fit for measures based on clinical observation, with INFIT and OUTFIT mean square statistics preferred within 0.5 and 1.7, and items with values <0.5 and >2.0 considered for removal.

Items were evaluated by Item Characteristics Curves, Category Probability Curves and, if possible, Differential Item Functioning analyses. If disordered thresholds between adjacent categories were found, collapsing or redefining response categories was considered [33].

Unidimensionality, the measurement of the single construct of ‘shoulder function’, was evaluated by Principal Component Analysis of residuals. The guidelines by Linacre and Tennant [37] proposed that an Eigenvalue of more than 2.0 indicates that a second construct might be present. If a second construct was significant and large, and represented by more than three items, deletion of items or splitting of items and construction of two related but different outcome measures was considered.

In WINSTEPS, reliability estimations are indicated by separation and reliability coefficients. The separation coefficient indicates the number of different ability groups a test can discriminate between [38]. The separation and reliability coefficient values correspond with one another. A reliability coefficient of 0.80 is similar to a separation coefficient of 2. This is a recommended reliability estimate for a test in any situation [33,38,39] and was set as a *minimum* for the SFInX. A reliability coefficient of 0.90 corresponds with a separation coefficient of 3, and was set as *preferred* reliability estimate for the SFInX.

Rasch analysis provides a person’s score on a logit scale from –∞ to + ∞. These scores were rescaled to a 0 to 100 interval-level scale to facilitate interpretation of SFInX scores.

## Results

Figure 1 provides an overview of each stage during development of the SFInX. Items from pilot testing to the final 13 items are presented in Table 2 along with the reason for exclusion where applicable.

**Table 2:** **SFInX items and exclusion criteria where applicable**

**Item**	**Reason of exclusion**
**Excluded during pilot testing**	
Get into bed	Not possible to judge the ability to perform this task
Lying flat, arms by the side	Not possible to judge the ability to perform this task
Get out of bed	Not possible to judge the ability to perform this task
Push up from chair	Not possible to judge the ability to perform this task
Push/slide chair forward	Consistency issues between testing locations
Reach with supporting hand	Did not measure ‘shoulder function’
**Excluded after pilot testing**	
Put on socks	Dressing requirements
Put on shoes	Dressing requirements
Put on jacket	Dressing requirements
Close zip on jacket	Dressing requirements
Keyboard	Lack of discrimination between people
Push off wall with 2 hands	Lack of discrimination between people
Lift object with arm by side	Redundant with item ‘hold object with arm by side’ included
Place item at shoulder level	Redundant with item ‘place object at shoulder level’ included
*Wash wall/window	Lacked appropriate simulation of target task
**Excluded prior to Rasch analysis**	
Arm swing in walking	Did not measure ‘shoulder function’
Lying on unaffected side	Lack of discrimination between people
Turn around in bed	Lack of discrimination between people
**Excluded based on findings from Rasch analysis**	
Light switch	Overfitting Rasch model, redundant
Hitch up trousers	Overfitting Rasch model, redundant
Place object above head with 2 hands	Overfitting Rasch model, contributing to potential second dimension
**Included items into final 13-item SFInX**	
1 drink from cup	
2 wash opposite armpit	
3 wash back of opposite shoulder	
4 comb hair	
5 tuck shirt into trousers	
6 wash lower back	
7 lying on affected side	
8 reach behind to get object	
9 hold object with arm by side	
10 carry heavy object with 2 hands	
11 place object at shoulder level	
12 hang up washing (sustained activity above head)	
13* throw ball overhead with 2 hands	

*Added items during pilot testing.

### Generation of items

Extraction of items from existing outcome measures used in studies of people with proximal humeral fracture resulted in 138 items.

Of the 32 individuals who agreed to participate, 15 clinicians (4 orthopaedic surgeons, 7 physical therapists, 4 occupational therapists) and 13 people with a proximal humeral fracture attended the focus groups. Characteristics of participants are detailed in Additional file 2: Table S1. The four focus groups generated 211 items.

After removal of duplicates, a total of 282 items from existing outcome measures and focus groups remained. Items were grouped as ‘self-care’, ‘cleaning/laundry’, ‘eating/kitchen’, ‘sleeping’, ‘in/around the house’, ‘car/driving/transport’, ‘leisure/sports/physical work’ and ‘other’. Lists of items are available from the authors upon request.

### Selection of items

Selection on pre-determined criteria and discussion resulted in 42 items included in the item rating questionnaire. Item ratings from 24 of 28 focus group participants (86%) were received. Thirty-two items were selected for pilot testing: 22 of the 25 items with an average score of ≥7/10 points on importance were selected, and ten items rated below 7/10 points were retained to ensure a diversity of activities and task difficulty.

During pilot testing (participant characteristics in Additional file 2: Table S2), two items reflecting a ‘sustained activity above the head’ were added since such tasks were lacking. A total of 34 items were defined and pilot tested, and 15 items were removed based on pilot testing (Table 2). These items had feasibility or judgement issues, did not reflect ‘shoulder function’ appropriately, were redundant or did not discriminate well among participants.

Prior to Rasch analysis, a further three items were removed due to testing issues: one item (‘arm swing in walking’) did not reflect ‘shoulder function’ adequately and two items (‘lying on unaffected side’ and ‘turn around in bed’) did not discriminate between people with little (performing no other tasks successfully) and regained ‘shoulder function’ (performing all other tasks successfully).

### Validation study participants

Ninety-two people with a proximal humeral fracture were recruited into the validation study (Table 3). The cause of fracture was a simple fall in 71 participants (77%), while a high energy trauma, such as a fall from a bicycle or horse, was the cause of fracture in 21 participants (23%). Nine people (10%) had sustained a fracture-dislocation which, after reduction, had a similar clinical course to an isolated proximal humeral fracture and were therefore included in the study. Home visits were made for 25 out of 92 (27%) initial assessments.

**Table 3:** **Descriptive characteristics of sample (n = 92)**

**Characteristics**		**no. (%) or Mean ± SD (range)**
Participants		92 (100%)
Men		13 (14%)
Women		79 (86%)
Age (years)		63.5 ± 13.9 (23–92)
Living situation		
Alone		23 (25%)
With spouse/family		69 (75%)
Time after fracture (weeks)		26.5 ± 15.1 (5–52)
1–3 months		20 (22%)
4–6 months		30 (33%)
7–9 months		19 (20%)
10–12 months		23 (25%)
Fracture side		
Right		42 (46%)
Left		50 (54%)
Fracture of dominant side		
Yes		44 (48%)
No		48 (52%)
Fracture management		
Conservative		74 (80%)
Surgical	ORIF	16 (17%)
	Hemi	2 (2%)
Fracture classifications		no. (%) per fracture type
AO classification	A	n = 53 (58%)
		1 (n = 19), 2 (n = 26), 3 (n = 8)
	B	n = 36 (39%)
		1 (n = 24), 2 (n = 11), 3 (n = 1)
	C	n = 3 (3%)
		1 (n = 1), 2 (n = 1), 3 (n = 1)
Neer classification	2-part	n = 55 (60%)
		2FD ant (n = 4) 2GT (n = 15),
		2aSN (n = 22), 2bSN (n = 9),
		2cSN (n = 5)
	3-part	n = 35 (38%)
		3FD ant (n = 2), 3GT (n = 31),
		3LT (n = 2)
	4-part	n = 2 (2%)
		4-part (n = 2)
Hertel classification		1 (n = 30), 2 (n = 1), 3 (n = 19),
		7 (n = 32), 8 (n = 1), 9 (n = 5),
		10 (n = 1), 12 (n = 3)

ORIF, open reduction internal fixation; Hemi, hemiarthroplasty; FD ant, anterior fracture dislocation; GT, greater tuberosity; SN, surgical neck; LT, lesser tuberosity.

### Rasch analysis and further item selection

Rasch analysis was performed with data on the 16 remaining SFInX items (1465 data points). Seven item responses (0.5%; 7/1472) were missing, as one participant, who was 5 weeks post-fracture, was unsure about the progress of fracture healing at that stage and did not want to attempt items involving weights and a ball.

**Figure 2:**
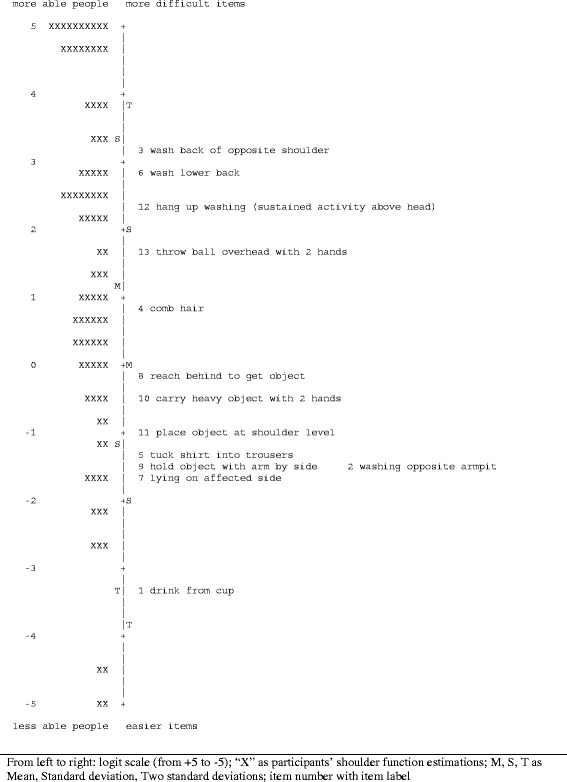
**13-item SFInX: item hierarchy and targeting.**

The GRSM and the PCM were run and WINSTEPS outputs regarding data fit to the models, item hierarchy, test targeting, unidimensionality and reliability estimations were analysed. Three items were removed at this point: items ‘light switch’, ‘hitch up trousers’ and ‘place object above head with 2 hands’ showed overfit to the models (Table 4) and were considered redundant since other items covered similar activities. Figure 2 is a graphical presentation of the linear measure of ‘shoulder function’ as defined by the 13 items including the item hierarchy (right) and the distribution of the sample (left).

**Table 4:** **Rasch item fit statistics of three overfitting and redundant items (comparison between GRSM and PCM)**

**GRSM**				**Item label**	**PCM**			
**INFIT**		**OUTFIT**			**INFIT**		**OUTFIT**	
**MNSQ**	**ZSTD**	**MNSQ**	**ZSTD**		**MNSQ**	**ZSTD**	**MNSQ**	**ZSTD**
0.58	−2.0	0.36	−0.9	hitch up trousers	0.68	−1.6	0.39	−0.8
0.48	−4.0	0.33	−2.1	place object above head	0.48	−3.9	0.33	−2.1
0.39	−1.5	0.05	−1.5	light switch	0.35	−1.6	0.05	−1.5

GRSM, Grouped Rating Scale Model; PCM, Partial-Credit Model; MNSQ, Mean Square; ZSTD, Standardised Z-score; SD, standard deviation.

From analyses with the PCM, four of the 13 remaining SFInX items (items 2, 8, 10 and 12) showed disordered thresholds and item 9 showed no difference between the participants’ average shoulder function when judged ‘unable’ or ‘partially able’ on this item. Therefore, the ‘partially able’ response categories of items 2, 8 and 9 were collapsed into one category ‘unable’. However, three response categories of items 10 and 12 were kept since in a larger sample the ‘partially able’ category might show clinical relevance for testing.

Unidimensionality testing using Principal Component Analysis of residuals showed that the main component ‘shoulder function’ explained 66.3% of the total variance in the 13 items. A potential second dimension (Eigenvalue 2.0) explained 5.3% of variance. Items ‘4. comb hair’ (loading 0.55),’ 12. hang up washing’ (loading 0.66) and ‘13. throw ball overhead with 2 hands’ (loading 0.60) were found to be the main items representing this component. However, comparison of person measures from positively and negatively loading items on this component showed a non-significant (*t* = 1.36, *p* = 0.18) mean difference of 0.68 logits (SE 0.50).

Comparing the results from both Rasch models, we chose the PCM as the best model to further develop the SFInX. Item and person fit (Table 5) was similar but slightly better with PCM, and changes made to items, in particular the collapsing of categories, were based on the PCM. Reliability estimations using this model indicated a person separation of 2.90 and person reliability coefficient of 0.89. The item separation was 6.10 and item reliability coefficient 0.97.

**Table 5:** **13-item SFInX: item fit and difficulty (ordered by misfit (INFIT MNSQ) of items to the Rasch model)**

**Item**			**PCM**			
**No.**	**Name**	**Difficulty**	**INFIT**		**OUTFIT**	
		**Logits (SE)**	**MNSQ**	**ZSTD**	**MNSQ**	**ZSTD**
6	wash lower back	2.85 (0.23)	1.53	2.6	1.41	1.0
7	lying on affected side	−1.61 (0.27)	1.43	2.1	1.28	0.7
3	wash back shoulder	3.20 (0.24)	1.30	1.6	1.17	0.5
10	carry heavy object	−0.47 (0.22)	1.28	1.2	1.49	0.8
2	wash armpit	−1.53 (0.38)	1.27	1.2	1.58	0.9
8	reach behind for object	−0.12 (0.32)	1.15	0.9	0.83	−0.2
9	hold object arm by side	−1.53 (0.38)	0.84	−0.7	0.44	−0.7
12	hang up washing	2.34 (0.21)	0.82	−0.9	0.58	−0.7
4	comb hair	0.83 (0.22)	0.79	−1.4	0.72	−1.2
5	tuck in shirt	−1.29 (0.27)	0.78	−1.4	0.65	−1.5
13	throw ball overhead	1.66 (0.21)	0.66	−2.1	0.46	−1.4
11	lift object shoulder level	−1.00 (0.35)	0.64	−2.0	0.35	−0.9
1	drink from cup	−3.34 (0.56)	0.60	−1.1	0.14	−1.0
	**Test mean**	**0.00 (0.30)**	**1.01**	**0.0**	**0.85**	**−0.3**
	**Test SD**	**1.93 (0.10)**	**0.31**	**1.6**	**0.46**	**0.9**

After Rasch analysis the SFInX contained 13 items to measure shoulder function, mainly targeting the ability to perform activities related to self-care and tasks around the house. Five items have two response categories: ‘unable’ and ‘able’. Eight items have a third response category ‘partially able’, which is chosen when compensatory movements are used to complete the task.

User-friendly scoring of the SFInX is important for easy interpretation. Based on the minimum and maximum person measures (0 points = −5.91 logits; 21 points = 6.06 logits), rescoring from a logit to 0–100 interval scale was achieved using the following formula derived from WINSTEPS:

$$ \mathrm{SFInX}\ \mathrm{score} = \left(\mathrm{logit}\ \mathrm{score}\ *\ 8.3580\right) + 49.38 $$

On the final SFInX clinical assessment form (Additional file 3) an easy rescoring table is presented allowing the total number of added raw points to be rescored to the SFInX score.

## Discussion

This study described the development of the SFInX, a unidimensional, interval-level scale of ‘shoulder function’ for people recovering from a proximal humeral fracture. It is the first clinician-observed outcome measure for ‘shoulder function’, where people are asked to perform tasks and are judged on their ability to complete the tasks.

The SFInX is a unidimensional index that focuses on the use of the affected arm to perform daily tasks. Many other shoulder outcome measures [14,40,41], such as the Constant Score [42] include related but distinct constructs such as pain and joint range of motion. People can function independently and satisfactorily with some range of motion restrictions or some degree of pain. Problems experienced by patients should be reflected in the scores when evaluating the shoulder. Hence, it is essential for measurement to target different constructs separately. The SFInX, as a unidimensional measure of ‘shoulder function’, is therefore likely to be used in conjunction with measures of other important outcomes, such as pain.

Three SFInX items were found to form a potential second dimension. Although the Eigenvalue was 2.0, the explained variance was only 5.3%. Also, differences in person ability estimations between items creating this potential dimension, and the remaining items were not significant. This would suggest that in the current data set a second dimension was not present. In further studies, however, it will be important to re-evaluate unidimensionality.

The SFInX as a clinician-observed outcome measure distinguishes itself from other types of administration such as purely performance-based [43,44], clinician-administered [15,42] or patient-reported measures [16,17]. Specific types of administration have their benefits and limitations, depending on the aim and focus of measurement. It is however important that high-quality, unidimensional tools are used for clinical measurement. Besides measuring the actual ability of a person to perform activities, patient-reported measures provide information on patient perception [45-47] and could therefore provide added value in the evaluation of people with a shoulder problem.

The final set of 13 SFInX items proposed in this study have undergone a rigorous selection process compared to other shoulder measures, and might be suitable to measure shoulder function in other populations. The items were developed through interviews with clinicians and patients, and through review of existing scales. This set of items might therefore be similar to components of other shoulder measures such as the DASH, Penn Shoulder score [48], and Simple Shoulder Test [49]. Item generation resulted in a list of 282 daily activities, which were systematically reduced during the selection process. An important selection method compared to other shoulder measures was Rasch analysis, which revealed redundant and not fitting items to the unidimensional construct of shoulder function.

Through these methods the SFInX has also potential for use in other populations than people recovering from a proximal humeral fracture. People suffering from a shoulder condition with a similar clinical picture, experiencing limitations in their daily activities might also benefit from functional measurements of the SFInX, but this would need to be confirmed with further study.

### Study limitations

Differential Item Functioning refers to the situation where items can function differently between subgroups of people with the same level of ability. Although, more informal analyses can be performed with a minimum of n = 30 per subgroup [28], more than 200 participants per subgroup is generally recommended [50]. With the current sample size of n = 92 only informal analyses were possible (no differential item functioning was found). Future analysis with more data should investigate differential item functioning in detail.

## Conclusion

The SFInX is a unidimensional, interval-level scale for people recovering from a proximal humeral fracture. It is a clinician-observed outcome measure for ‘shoulder function’ that is intended for use in clinical practice and research^a^. To evaluate the potential of the SFInX to monitor the functional progress of individuals and in clinical trials of treatment for people with a proximal humeral fracture, further psychometric evaluation that includes reliability and longitudinal validity testing is required.

## Endnote

^a^The SFInX is freely available to use. Through La Trobe University, it has been made possible to create a webpage for the SFInX: http://SFInX.blogs.latrobe.edu.au. The webpage mainly focusses on free training and instructions to use the SFInX, and the SFInX measurement properties. Updates on the SFInX, current and future projects will be posted on the webpage and blog. We kindly request that if there are plans to use the SFInX in a clinical setting or research project, an email to the first and corresponding author will be sent to notify us of the intention: a.vandewater@latrobe.edu.au. If the SFInX was used in a project, we kindly request to cite the instrument by citing this publication.

## Additional files

Additional file 1:
**SFInX manual – clinical assessment form and instructional manual.**
Additional file 2: Table S1. Focus group participant characteristics. **Table S2.** Pilot-test sample characteristics. Additional file 3:
**Shoulder Function Index – clinical assessment form.**

## Abbreviations

DASH: Disabilities of the arm, shoulder and hand
GRSM: Grouped-rating scale model
PCM: Partial credit model
SFInX: Shoulder function index
