# Supplementary material for: The Shoulder Function Index (SFInX): a clinician-observed outcome measure for people with a proximal humeral fracture
Source: BMC Musculoskelet Disord. 2015 Feb 18;16:31. doi: 10.1186/s12891-015-0481-x (PMC4336677; doi:10.1186/s12891-015-0481-x)
Supplement: Additional file 1: — SFInX manual – clinical assessment form and instructional manual. [file 12891_2015_481_MOESM1_ESM.pdf]

Shoulder Function Index (SFInX)

clinical assessment form and
instructional manual

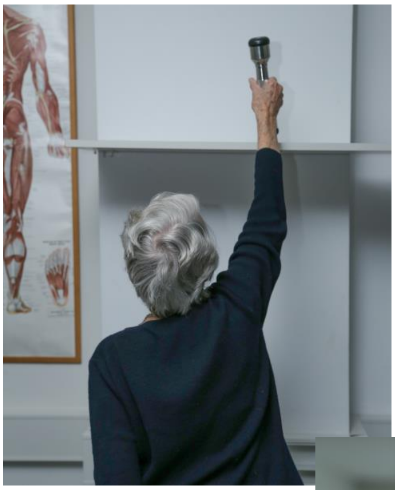

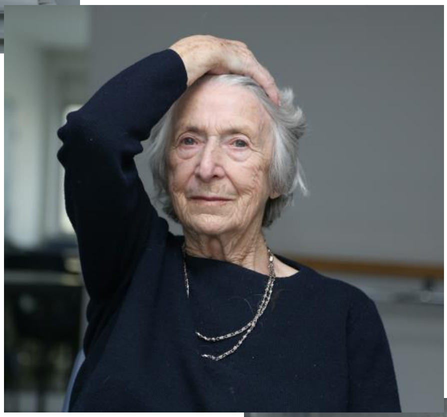

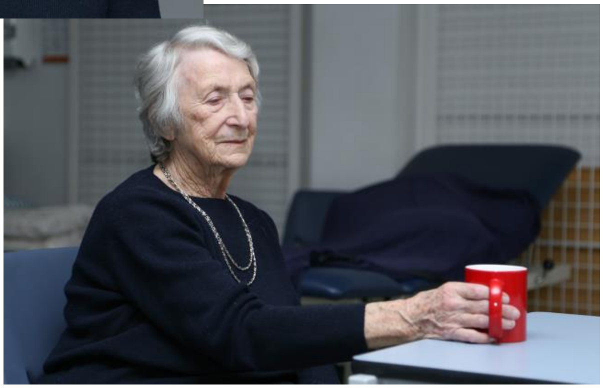

When citing the SFInX, please refer to one of the SFInX development articles rather than this manual, citing at least the development article published in 2015 in BMC Musculoskeletal Disorders titled "The Shoulder Function Index (SFInX): a clinician-observed outcome measure for people with a proximal humeral fracture"

Alexander TM van de Water
Megan Davidson
Nora Shields
Matthew C Evans
Nicholas F Taylor
© 2013

Contact: Alexander TM van de Water, a.vandewater@latrobe.edu.au
Website: http://SFInX.blogs.latrobe.edu.au

The development of the SFInX has been supported by La Trobe University, Victoria, Australia

Table of Contents

Shoulder Function IndeX - clinical assessment form ____ 4

Shoulder Function Index - hierarchy of SFInX items ____ 5

Descriptions of SFInX items ____ 6

ITEM 1: drinking from a cup ____ 7

ITEM 2: washing opposite armpit ____ 8

ITEM 3: washing back opposite shoulder ____ 9

ITEM 4: combing hair/running hand through hair ____ 10

ITEM 5: tucking shirt into pants ____ 11

ITEM 6: washing lower back ____ 12

ITEM 7: lying on affected side ____ 13

ITEM 8: reaching behind to get an object ____ 14

ITEM 9: holding an object for a longer period ____ 15

ITEM 10: carrying a heavy object with two hands ____ 16

ITEM 11: placing an object on a shelf at shoulder level ____ 17

ITEM 12: moving an object above head level ____ 18

ITEM 13: throwing a ball with two hand over-head ____ 19

Recommendations for items requiring equipment ____ 20

SFInX items linked to the ICF ____ 21

Shoulder Function IndeX - clinical assessment form

SFInX score:

Patient name: ____

Time after injury (in weeks): __________   Side of injury: R / L

Date of assessment: ___ / ___ / ______

**Items**		**Scores**		
		**0**	**1**	**2**
1	Drinking from a cup	☐ Unable	☐ Able	
2	Washing the opposite armpit	☐ Unable	☐ Able	
3	Washing the back of the opposite shoulder	☐ Unable	☐ Partially able	☐ Able
4	Combing hair	☐ Unable	☐ Partially able	☐ Able
5	Tucking shirt into pants	☐ Unable	☐ Partially able	☐ Able
6	Washing lower back	☐ Unable	☐ Partially able	☐ Able
7	Lying on the affected side	☐ Unable	☐ Partially able	☐ Able
8	Reaching behind to get an object	☐ Unable	☐ Able	
9	Holding an object for a longer period	☐ Unable	☐ Able	
10	Carrying a heavier object with two hands	☐ Unable	☐ Partially able	☐ Able
11	Placing an object on a shelf at shoulder level	☐ Unable	☐ Able	
12	Sustaining activity above head	☐ Unable	☐ Partially able	☐ Able
13	Throwing a ball with two hands over-head	☐ Unable	☐ Partially able	☐ Able
**Total number of points (raw score)**		0	+	+ =

Conversion from raw score to SFInX score

Raw score	0	1	2	3	4	5	6	7	8	9	10	11	12	13	14	15	16	17	18	19	20	21
SFInX score	0	12	20	27	32	36	40	43	46	49	52	55	58	61	64	67	70	73	77	81	89	100

Shoulder Function Index - hierarchy of SFInX items

**HARDEST**	ITEM 3	washing back opposite shoulder
↑	ITEM 6	washing lower back
	ITEM 12	moving an object above head level
	ITEM 13	throwing a ball with two hands overhead
	ITEM 4	combing hair/running hand through hair
	ITEM 8	reaching behind to get an object
	ITEM 10	carrying a heavy object with two hands
	ITEM 11	placing an object on a shelf at shoulder level
	ITEM 5	tucking shirt into pants
	ITEM 9	holding an object for a longer period
	ITEM 2	washing opposite armpit
↓	ITEM 7	lying on affected side
**EASIEST**	ITEM 1	drinking from a cup

Descriptions of SFInX items

ITEM 1: drinking from a cup

Description	The ability to lift the cup to the mouth to drink, and to place the cup back is being judged.
Equipment	cup
Position	sitting
Instruction (clinician)	place a cup without handles (or instruct not to use the handles) in front of the person to be tested.
Instruction (patient)	while keeping your head in an upright position, reach and pick up the cup with the hand of the affected arm. Move the cup to your mouth to drink, then place the cup back.

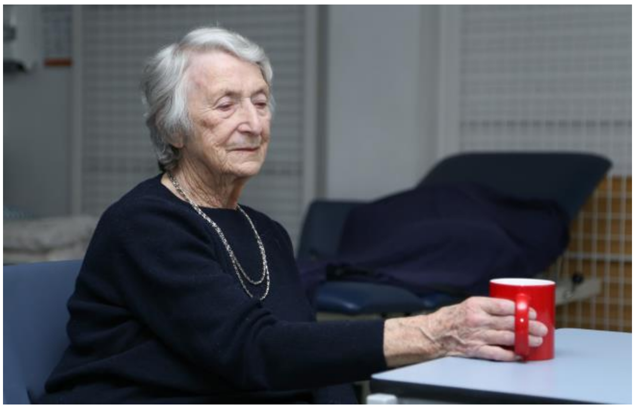

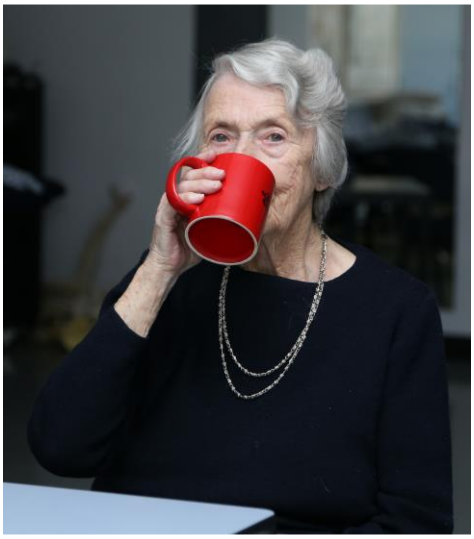

Decision rules	**Able**	reaches for the cup with the hand of the affected arm, picks up the cup, moves the cup to the mouth and places the cup back to starting position; affected arm is held off the body; movements of the affected arm are unsupported
	**Unable**	cannot take the cup to the mouth to drink; or less (for example, unable to reach or pick up cup)

ITEM 2: washing opposite armpit

Description	The ability to simulate washing of the opposite armpit is being tested
Equipment	—
Position	sitting
Instruction (patient)	reach with the hand of the affected arm to the armpit of the opposite arm and apply pressure while moving the hand of the affected arm backward and forward, up and down

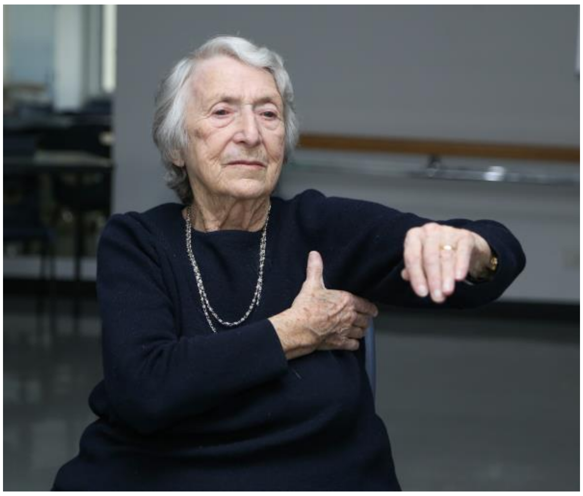

Decision rules	**Able**	reaches, applies pressure and is able to move the palm of the hand of the affected arm in all directions under the armpit of the opposite side; movements of the affected arm should be unsupported; comparison of hand positioning with the unaffected side is recommended
	**Unable**	cannot reach the opposite armpit equally far as the unaffected arm (reaching with fingers only is not sufficient to 'wash' the opposite armpit)<br><br>OR<br><br>reaches, but cannot apply pressure and/or make movements with the hand of the affected arm

ITEM 3: washing back opposite shoulder

Description	The ability to simulate washing of the back of the opposite shoulder is being tested
Equipment	—
Position	sitting
Instruction (patient)	reach with the hand of the affected arm across the body to the back the opposite shoulder and apply pressure while moving the hand of the affected arm sideways

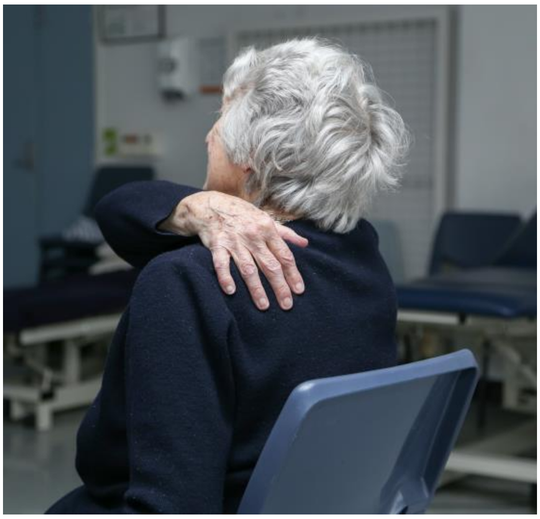

Decision rules	**Able**	reaches, applies pressure and moves the hand of the affected arm sideways; movements of the affected arm should be unsupported; comparison of hand positioning with the unaffected side is recommended
	**Partially Able**	reaches equally far as the unaffected arm, but cannot apply pressure and/or make the required movements to the sides with the hand of the affected arm
	**Unable**	cannot reach the back of the opposite shoulder equally far as the unaffected arm (for example, reaches top of opposite shoulder only)

ITEM 4: combing hair/running hand through hair

Description	The ability to simulate combing hair (or running hand through hair) with the affected arm is being tested
Equipment	—
Position	sitting
Instruction (patient)	while keeping your head in an upright position, move the fingers and palm of the hand of the affected arm from your forehead over the top of the head into your neck

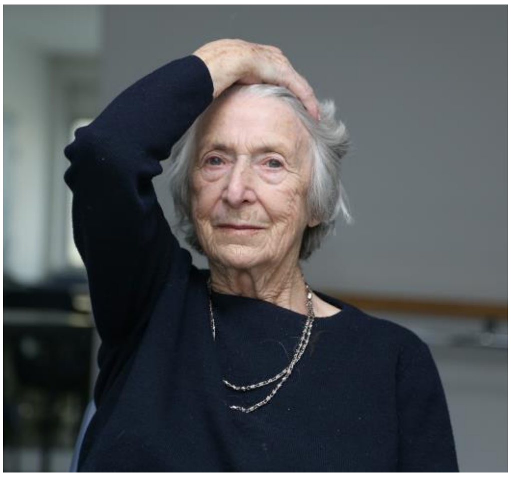

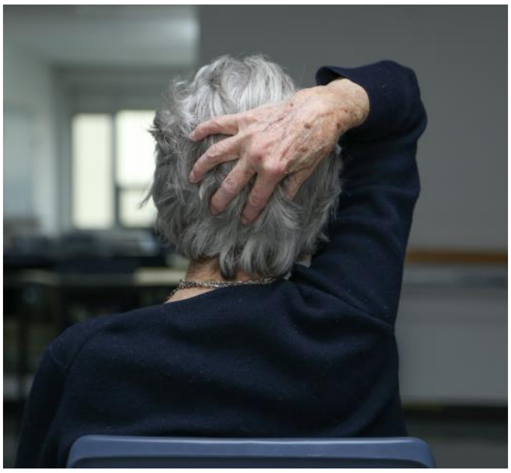

Decision rules	**Able**	moves fingers and palm of the hand of affected side from the mid-forehead into the neck; movements of the affected arm are unsupported; head is held in upright position
	**Partially Able**	moves fingers and palm of the hand of affected side from the mid-forehead through hair to the top of the head; unable to move over the top of the head and/or moves the hand from the top of the head to behind the ear/side of the head; head is held in upright position
	**Unable**	cannot move fingers and palm of the hand of affected side to the top of the head; or less

ITEM 5: tucking shirt into pants

Description	The ability to tuck in shirt (or similar) into pants (or similar) in a standing position is being tested
Equipment	—
Position	standing
Instruction (patient)	tuck your shirt into the pants using the hand of the affected arm on the affected side

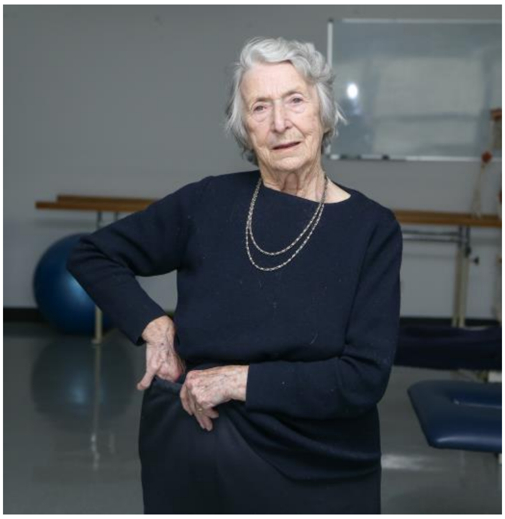

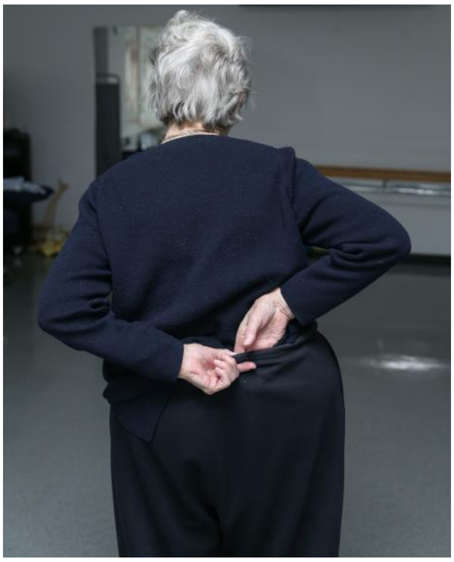

Decision rules	**Able**	uses the hand of the affected arm to tuck in the shirt on the affected side at the front (from zip/middle), the side and the back (to spine/middle)
	**Partially Able**	uses the hand of the affected arm to tuck in the shirt on the affected side at the front and the side
	**Unable**	uses the hand of the affected arm to tuck in the shirt on the affected side at the front only; or less

ITEM 6: washing lower back

Description	The ability to simulate washing of the lower back (lumbar spine area) is being tested
Equipment	—
Position	standing
Instruction (patient)	move the palm of the hand of the affected arm to your lower back. Apply pressure while moving the hand in all directions over your lower back

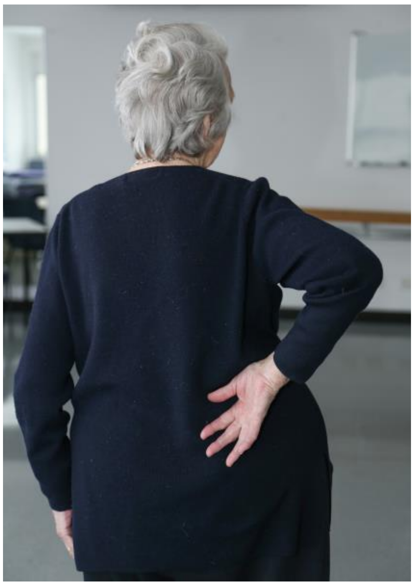

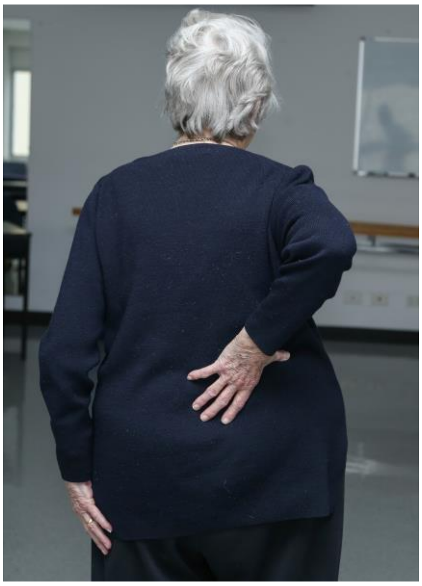

Decision rules	**Able**	reaches the lower back with the hand of the affected side; is able to put the palm of the hand flat onto the lower back while applying pressure and moving the hand in all directions (up, down, right, left) over the lower back
	**Partially Able**	reaches the lower back with the hand of the affected side; and puts the palm of the hand flat onto the lower back; unable to move the hand
	**Unable**	cannot put the palm of the hand of the affected arm flat onto the lower back; or less (for example, cannot reach the lower back with the hand of the affected side)

ITEM 7: lying on affected side

Description	The ability to lie on the affected side with the unaffected arm in front of the body is being tested. Use a single pillow for head support in lying position
Equipment	bed
Position	lying
Instruction (patient)	lie on the affected side with the unaffected arm in front of your body

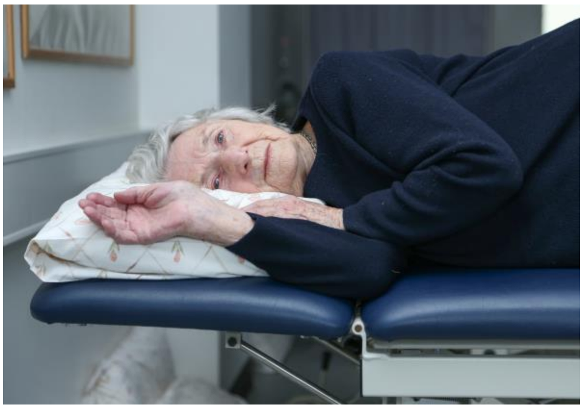

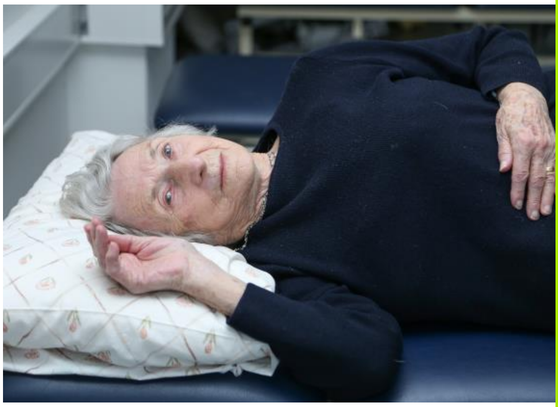

Decision rules	**Able**	lies on the affected side; that is, directly on top of the affected shoulder, with the unaffected arm in front of and across the body
	**Partially Able**	lies partially on the affected side; that is, on the scapula rather than the head of the humerus, with the unaffected arm in front of and across the body
	**Unable**	does not lie on the affected side

ITEM 8: reaching behind to get an object

Description	The ability to reach behind (for example, to back seat in a car) to pick up an object is being tested
Equipment	object (for example, heavy book, bottle; approximately 1.5 kg)
Position	sitting; with the back supported by the backrest of the chair
Instruction (clinician)	the chair on which the person to be tested will be seated, has to be positioned in front of a second chair or plinth. The object is placed on the second chair or plinth. The object on the second chair or plinth is approximately one arm's length behind the person
Instruction (patient)	reach for and pick up the object on the chair behind you with the hand of the affected arm, bring it forward, and place it onto your lap

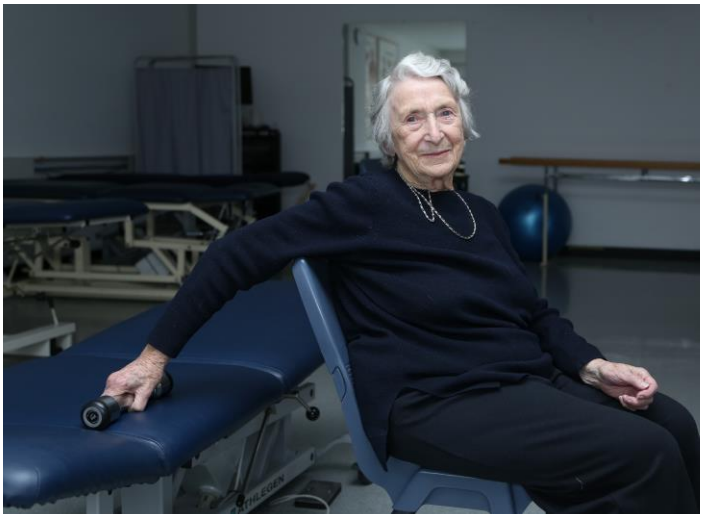

Decision rules	**Able**	reaches behind for the object; picks up the object and brings the object forward onto lap using the hand of the affected arm.<br>The strategy chosen is not to be judged (for example, internal/external rotation of the shoulder)<br>The person must remain seated in approximately the starting position; that is, with back supported by the backrest of the chair with the legs forward. Rotating away from the backrest by a large amount from the backrest is not permitted
	**Unable**	reaches behind for the object, but unable to bring the object forward (for example, due to movement required or weight of object); or unable to reach behind for the object

ITEM 9: holding an object for a longer period

Description	The ability to hold an object (for example, shopping bag) for a longer period
Equipment	object (for example, shopping bag with contents; approximately 3 kg)
Position	standing
Instruction (patient)	lift up and then hold the object for 30 seconds in the hand of the affected side with you arm by you side

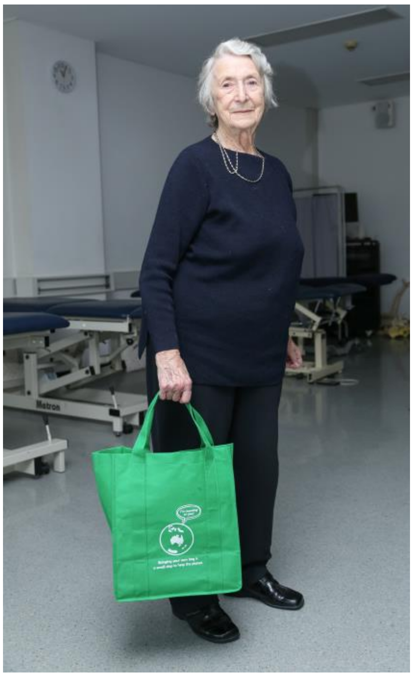

Decision rules	**Able**	lifts and holds the object for 30 seconds; affected arm is held off the body, the affected arm is unsupported
	**Unable**	lifts and holds the object for <30 seconds; or unable to lift the object

ITEM 10: carrying a heavy object with two hands

Description	The ability to carry/hold heavier items with two hands is being tested
Equipment	object (for example, a full box, weights; approximately 6 kg)
Position	standing and walking
Instruction (clinician)	person to be tested starts with approximately 90 degrees of elbow flexion; tester provides the person to be tested carefully with the object
Instruction (patient)	hold the object with both hands with elbows at right angles. Then, walk 5 metres forward, turn around and walk forwards back to the starting position

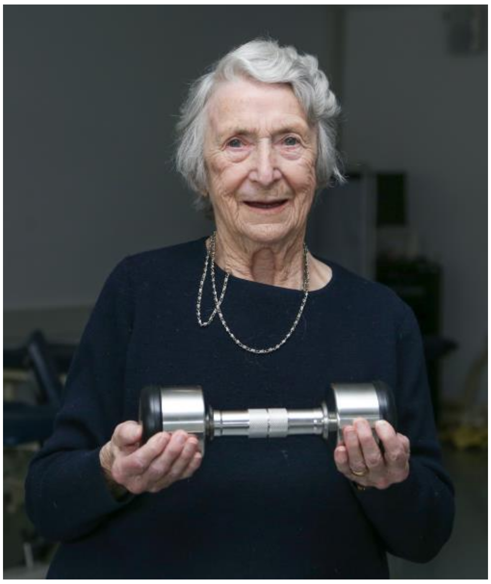

Decision rules	**Able**	holds the object equally in two hands; walks forward, turns and walks back without compensation in holding the object
	**Partially Able**	holds the object with two hands, but compensates so that the unaffected side is carrying more of the weight (for example, by changing position of the object in the hands); or needs to rest/pause
	**Unable**	holds the object for short period only; or less

ITEM 11: placing an object on a shelf at shoulder level

Description	The ability to lift and then place an object on a shelf (or similar) at shoulder level is being tested
Equipment	object (for example, book, bottle; approximately 1.5 kg)
Position	standing; one arm's length from shelf
Instruction (patient)	place the object on the shelf at shoulder level using the hand of your affected arm

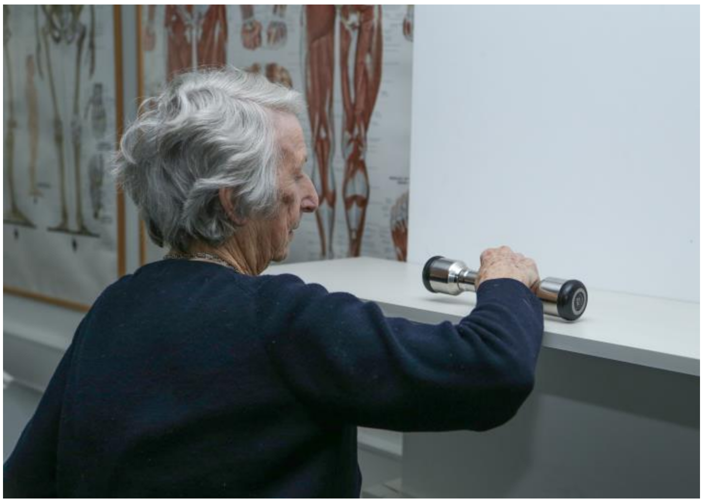

Decision rules	**Able**	lifts and places the object on the shelf at shoulder level; movements of the affected arm are unsupported at all times<br>Large compensation by standing on toes, or leaning backward (extension in upper body) with the aim to raise the object is not permitted
	**Unable**	lifts the object but not high enough to place on the shelf at shoulder level; or less

ITEM 12: moving an object above head level

Description	The ability to perform a prolonged activity above the head, such as hanging up washing or moving books/plates from one side to another on a shelf above the head, is being tested
Equipment	object (for example, book, bottle; approximately 1.5 kg)
Position	standing
Instruction (clinician)	before instructing the person to be tested, the tester is to place the object on a shelf above the head, approximately in line with the position of the person's left shoulder
Instruction (patient)	take the object in the hand of the affected arm, lift the object off the shelf and move the object from left (shoulder width) to right (shoulder width) and from right to left, and repeat this five times. Try not to rest the arm by putting the object down on the shelf

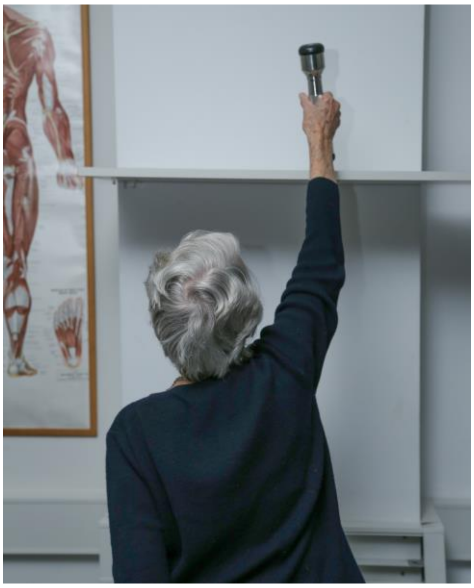

Decision rules	**Able**	takes and lifts the object off the shelf; maintains this forward flexion-elevation position while making movements sideways ten times (5x left-to-right-to-left) without rests
	**Partially Able**	takes and lifts the object off the shelf; moves the object sideways for less than ten times before needing to rest (for example, by placing the object on the shelf)
	**Unable**	takes and lifts the object off the shelf only; or unable to lift the object but moves (slides) it sideways on the shelf; or less (for example, cannot reach for the object)

ITEM 13: throwing a ball with two hand over-head

Description	The ability to throw a ball overhead using two hands is being tested
Equipment	ball (for example, soccerball or basketball)
Position	standing
Instruction (clinician)	provide the person to be tested with a ball<br><br>ensure that the person to be tested holds the ball in front of their body at waist level with two hands (starting position).<br>Stand approximately 5 metres in front of the person to be tested
Instruction (patient)	bring the ball with two hands over and behind the head, into your neck. Then, throw the ball forward (by bringing the ball back over the head, then forward) to the tester

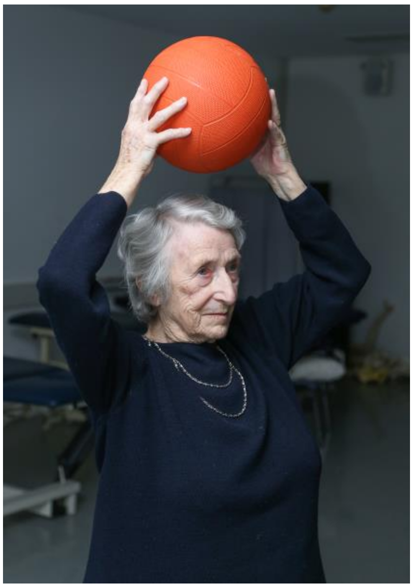

Decision rules	**Able**	makes the movements as required; controls the ball with both hands; and creates a forward throw to tester approximately 5 metres away
	**Partially Able**	makes the movements as required; controls the ball with both hands; then,<br>creates a forward throw, but is unsuccessful in distance (balls drops) or direction (not to tester) OR compensates clearly by flexion of the head<br>OR<br>brings the ball straight above the head or further back (but not into the neck); controls the ball with both hands; and has a successful throw in both distance and direction
	**Unable**	makes the movements as required only or less (for example, unable to make required movements); unable to make a throwing action

Recommendations for items requiring equipment

Chair	Items in which the position is “sitting” have to be performed in a standardised chair without armrests and without rollers. The person to be tested should have the feet on the floor, and the back supported by the backrest. In case testing is performed at a person’s home, a kitchen chair is often sufficient and recommended.

Item 1

Cup	A cup (no use of handles), mug or little water bottle containing some water or of some weight is recommended. A person has to be able to, or show they are to, drink from the cup.

Item 7 (and 8)

Bed	A hospital bed or plinth which is adjustable in height, or a person’s own bed at home should be used for testing. A single pillow should be used for comfort. Additional pillows might be needed for a person’s comfort in lying position if the person has a certain condition e.g. apnea.

Items 8, 11 and 12

Object 1.5 kg	Use of a heavy book, 1.5L bottle water, weight or other object, which weights or approximates 1.5 kg and which is able to be held comfortably in one hand is recommended.

Item 9

Bag + Object 3kg	A small box, two 1.5L bottles water, weight or other object which weights or approximates 3 kg, placed in a (shopping) bag is recommended.

Item 10

Object 6kg	Use of a pile of heavy books, a box, weight(s) or other object(s) which weight(s) or approximate(s) 6 kg, and can be held in two hands (with shoulders in neutral, elbows in 90 degrees flexion, forearms neutral or supinated) is/are recommended.

Item 13

Ball	Use of a soccer/football (or similar) is recommended.

SFInX items linked to the ICF

item		ICF code/category	Additional information
1.	Drink from a cup	d4300 Lifting<br>d560 Drinking	cup without handles
2.	Washing the opposite armpit	d5100 Washing body parts	opposite armpit
3.	Washing the back of the opposite shoulder	d5100 Washing body parts	back of opposite shoulder
4.	Combing hair	d5202 Caring for hair	combing, running hand through hair
5.	Tucking shirt into pants	d5400 Putting on clothes	tucking shirt
6.	Washing lower back	d5100 Washing body parts	above your head
7.	Lying on the affected side	d4150 Maintaining a lying position	on the affected side
8.	Reaching behind to get an object	d4452 Reaching<br>d4300 Lifting	
9.	Holding an object for a longer period	d4301 Carrying in the hands<br>d6200 Shopping	shopping bag
10.	Carrying a heavier object with two hands	d4301 Carrying in the hands<br>d4600 Walking around within the home	heavy object
11.	Placing an object on a shelf at shoulder level	d4300 Lifting	
12.	Sustaining activity above head (e.g. hanging up washing)	d4300 Lifting<br>d6400 Washing and drying clothes and garments	
13.	Throwing a ball with two hands over-head	d4454 Throwing<br>d4300 Lifting	ball with two hands

Photography by: Alice Hill
Model: Patricia Giannarelli
