# Supplementary material for: The Shoulder Function Index (SFInX): a clinician-observed outcome measure for people with a proximal humeral fracture
Source: BMC Musculoskelet Disord. 2015 Feb 18;16:31. doi: 10.1186/s12891-015-0481-x (PMC4336677; doi:10.1186/s12891-015-0481-x)
Supplement: Additional file 2: Table S1. — Focus group participant characteristics. Table S2. Pilot-test sample characteristics. [file 12891_2015_481_MOESM2_ESM.pdf]

Additional file 2

Table S1 | Focus group participant characteristics

Patients (n=13)*			Clinicians (n=15)*		
Gender	women	10 (77%)	Gender	women	8 (53%)
	men	3 (23%)		men	7 (47%)
Age (years)		64.5 ± 12.3 (44-86)	Age (years)		39.2 ± 8.8 (25-53)
Time post-fracture (in weeks)		25.9 ± 9.8 (10-41)	Profession		
			orthopaedic surgeon		4 (27%)
Fracture side	Right	6 (46%)	physiotherapist		7 (47%)
	Left	7 (54%)	occupational therapist		4 (27%)
Treatment	ORIF	2 (15%)	Experience (years)		
	Conserv	11 (85%)	orthopaedic surgeon		10.8 ± 7.7 (5-22)
			physiotherapist		21.0 ± 8.4 (10-31)
			occupational therapist		6.5 ± 2.7 (4-10)

* Values are no. (%) or Mean ± SD (range)
ORIF, Open Reduction Internal Fixation; Conserv, Conservative management

Table S2 | Pilot-test sample characteristics (n=12)*

Gender	women	11 (92%)
	men	1 (8%)
Age (years)		71.0 ± 10.4 (range 50-86)
Time post-fracture (days)		162.2 ± 101.2 (range 50-359)
Fracture side	Right	4 (33%)
	Left	8 (67%)
AO Classification	A	1 (n=4), 3 (n=2)
	B	1 (n=6)
Neer Classification		2GT (n=4), 3GT (n=4)
		3LT (n=1)
		2aSN (n=1), 2bSN (n=2)
Hertel Classification		1 (n=2), 3 (n=4), 7 (n=5)
		10 (n=1)

*Values are mean ± SD (range) or number (%) or (number)
GT, greater tuberosity; LT, lesser tuberosity; SN, surgical neck
