# Supplementary material for: The Shoulder Function Index (SFInX): a clinician-observed outcome measure for people with a proximal humeral fracture
Source: BMC Musculoskelet Disord. 2015 Feb 18;16:31. doi: 10.1186/s12891-015-0481-x (PMC4336677; doi:10.1186/s12891-015-0481-x)
Supplement: Additional file 3: — Shoulder Function Index – clinical assessment form. [file 12891_2015_481_MOESM3_ESM.pdf]

Shoulder Function Index - clinical assessment form

SFInX score:

Patient name: ____

Time after injury (in weeks): _________ Side of injury: L / R

Date of assessment: ___ / ___ / ______

Items		Scores		
		0	1	2
1	Drinking from a cup	☐ Unable	☐ Able	
2	Washing the opposite armpit	☐ Unable	☐ Able	
3	Washing the back of the opposite shoulder	☐ Unable	☐ Partially able	☐ Able
4	Combing hair	☐ Unable	☐ Partially able	☐ Able
5	Tucking shirt into pants	☐ Unable	☐ Partially able	☐ Able
6	Washing lower back	☐ Unable	☐ Partially able	☐ Able
7	Lying on the affected side	☐ Unable	☐ Partially able	☐ Able
8	Reaching behind to get an object	☐ Unable	☐ Able	
9	Holding an object for a longer period	☐ Unable	☐ Able	
10	Carrying a heavier object with two hands	☐ Unable	☐ Partially able	☐ Able
11	Placing an object on a shelf at shoulder level	☐ Unable	☐ Able	
12	Sustaining activity above head	☐ Unable	☐ Partially able	☐ Able
13	Throwing a ball with two hands over-head	☐ Unable	☐ Partially able	☐ Able
**Total number of points (raw score)**		0	+	+ =

Conversion from raw score to SFInX score

Raw score	0	1	2	3	4	5	6	7	8	9	10	11	12	13	14	15	16	17	18	19	20	21
SFInX score	0	12	20	27	32	36	40	43	46	49	52	55	58	61	64	67	70	73	77	81	89	100

© 2013 Alexander T.M. van de Water
